# Supplementary material for: A primordial germ cell-like-cell platform enables CRISPRi screen for epigenetic fertility modifiers
Source: EMBO Rep. 2025 Nov 13;26(23):6044–78. doi: 10.1038/s44319-025-00633-z (PMC12678824; doi:10.1038/s44319-025-00633-z)
Supplement: Supplementary file 1 — Appendix [file 44319_2025_633_MOESM1_ESM.pdf]

Appendix for “A primordial germ cell-like-cell platform enables CRISPRi screen for epigenetic fertility modifiers”

Table of Contents

Appendix Figure S1. Reprogramming properties of 4TF-induced PGCLCs 2

Appendix Figure S2. FACS gating strategy for the isolation of PGCLCs (Stella-GFP+) from EBs.........3

Appendix Figure S3. Neurodevelopment pathways suppressed in 4TF-induced PGCLCs 4

Appendix Figure S4. Heatmaps showing the average GSVA enrichment score of selected germ cell development pathways in Ck- and 3TF- induced PGCLCs 5

Appendix Figure S5. Top 10 enriched biological process GO terms in Ck-, 3TF-, Nanog- and 4TF-induced PGCLCs identified by GSEA..6

Appendix Figure S6. Volcano plot comparing transcriptomes of Ck-, 3TF-, Nanog- and 4TF-induced PGCLCs to ESC 7

Appendix Figure S7. STRING analysis of upregulated DEGs in PGCLCs compared to ESCs..............8

Appendix Figure S8. Differentiation of Nanog-induced PGCLCs 9

Appendix Figure S9. Differentiation of 4TF-induced PGCLCs in adherent cell culture plates 10

Appendix Figure S10. Library construction 11

Appendix Figure S11. CRISPRi library characterization 12

Appendix Figure S12. Phenotypic analysis of female pups 13

Appendix Figure S13. Absence of apoptotic cells in atrophic seminiferous tubules 14

Appendix Figure S14. Impact of FGFRi on differentiation of 4TF-induced PGCLCs from formative ESCs 15

Appendix Figure S15. Overexpression of Nanog in suspended formative ESCs 16

Appendix Figure S16. Comparation features of 4TF-inducible system with current available PGCLC differentiation systems 17

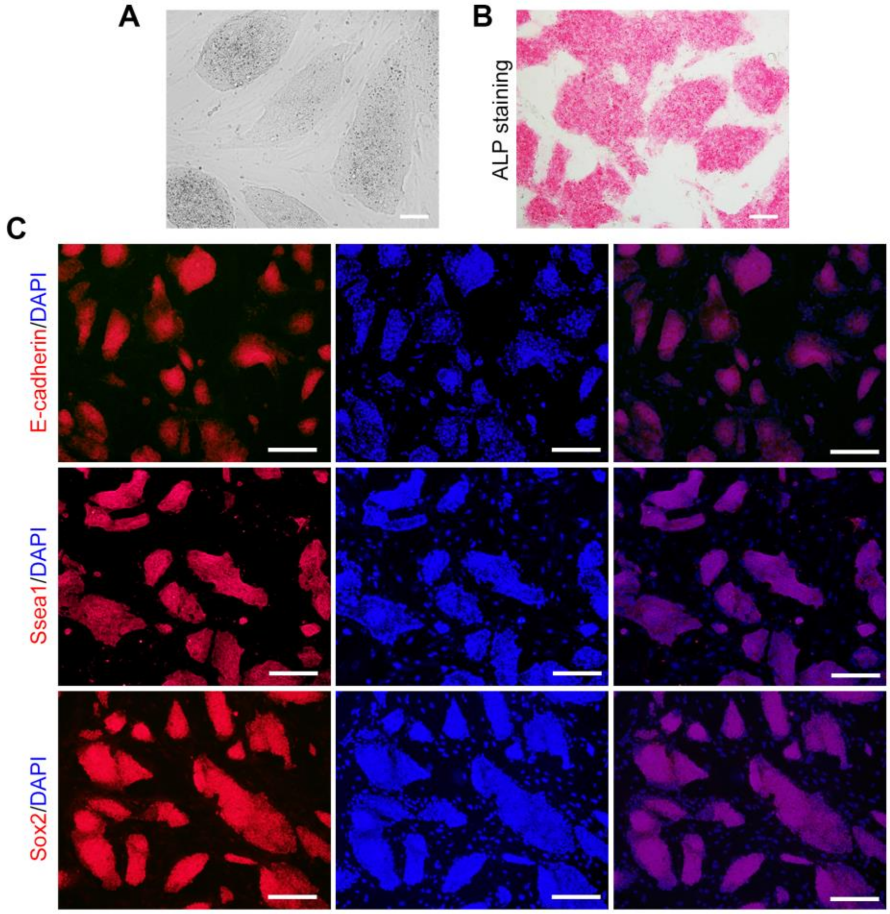

Appendix Figure S1. Reprogramming properties of 4TF-induced PGCLCs. A. Morphology of EGCs derived from 4TF-induced PGCLCs (Stella-eGFP$^+$ cells) at day 6. B. ALP (alkaline phosphatase) staining of EGCs. C. IF of E-cadherin, SSEA1 and SOX2 in EGCs. Scale bars in A and B = 50μm and in C = 100μm.

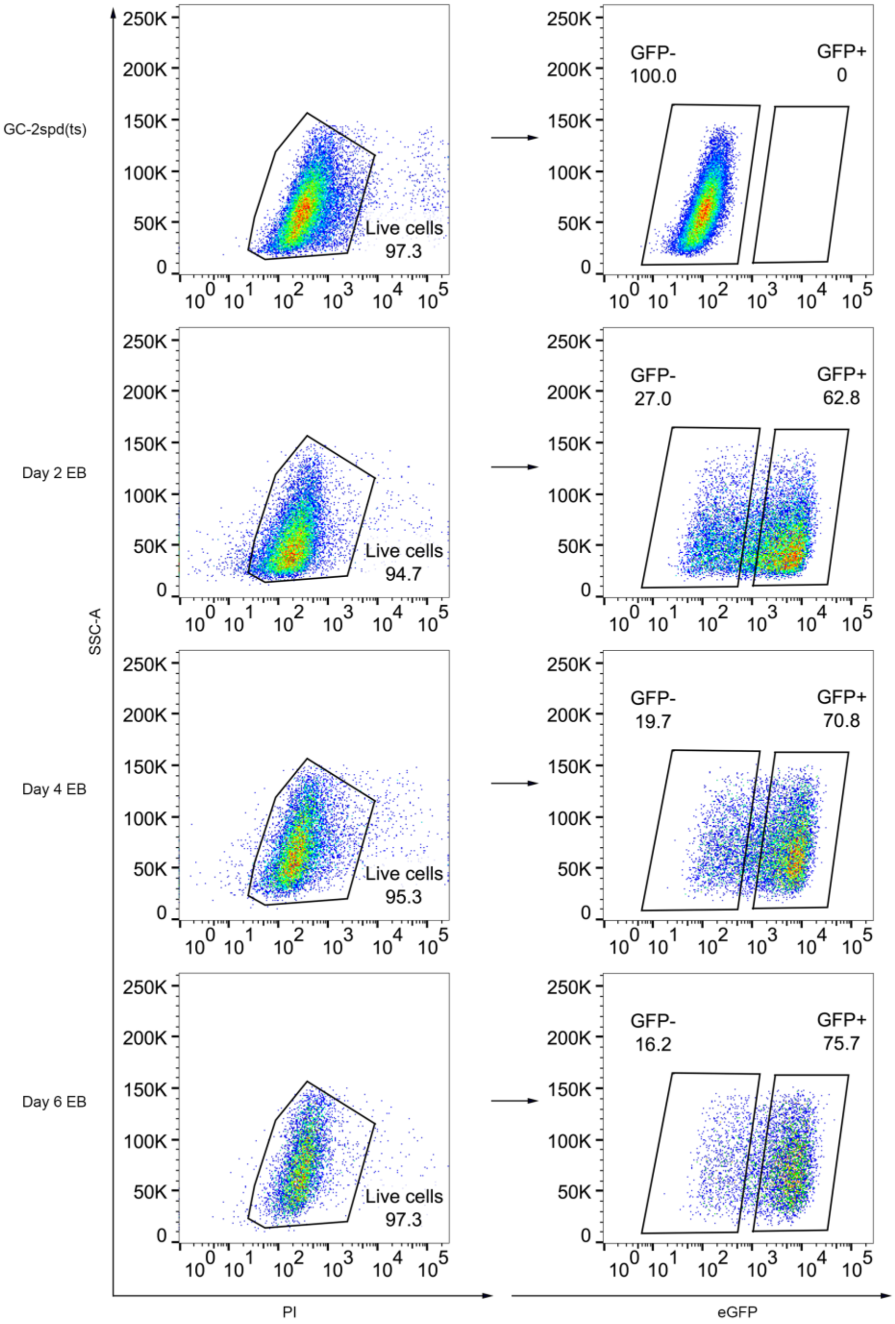

Appendix Figure S2. FACS gating strategy for the isolation of PGCLCs (Stella-GFP+) from EBs. GC-2spd(ts) cells served as GFP$^-$ reference. Gating for live cells was performed using propidium iodide (PI).

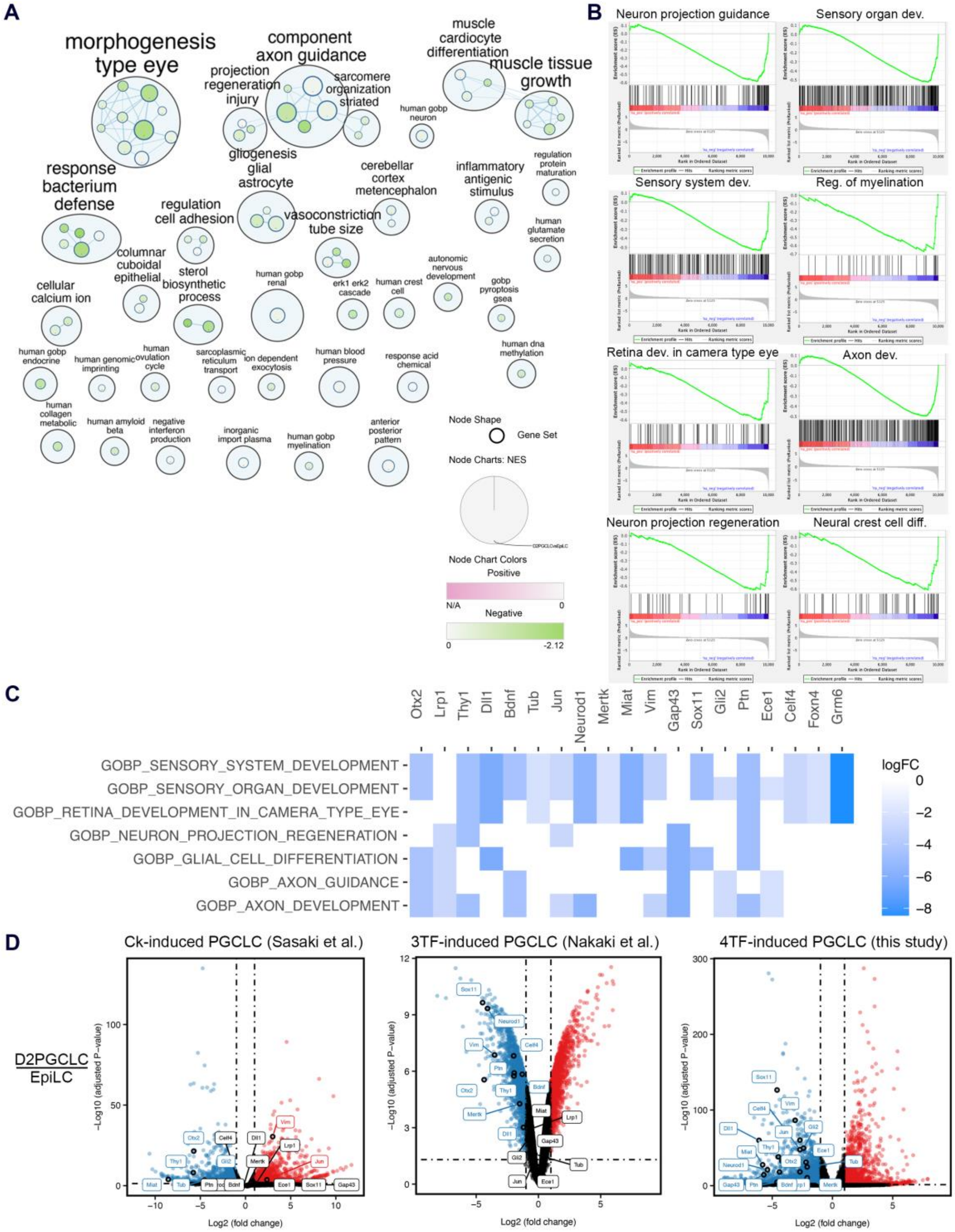

Appendix Figure S3. Neurodevelopment pathways suppressed in 4TF-induced PGCLCs. A. Network map illustrating enrichment of GOBP (Gene Ontology Biological Process) in EpiLCs vs. D2PGCLCs. Gene sets (nodes) are connected based on shared edges. Nodes downregulated in EpiLCs are highlighted in green. The size of each node corresponds to the percentage of enriched genes in the set, and color intensity indicates the degree of expression change. Blue lines represent shared gene overlap between sets. Clusters of gene sets with related functions are circled and labeled to emphasize prevalent biological functions. B. Suppression of neurodevelopmental processes in EpiLCs vs. D2PGCLCs. C. Heatmap of GO enrichment analysis for a subset of genes related to neurodevelopment. Displayed are log2 fold change (logFC) values between D2PGCLCs and EpiLCs. White indicates no association with the corresponding pathway. D. Volcano plot comparing transcriptomes of Ck-, 3TF- and 4TF- induced PGCLCs to EpiLCs. Dashed lines represent significance thresholds. Selected genes involved in neurodevelopment are highlighted.

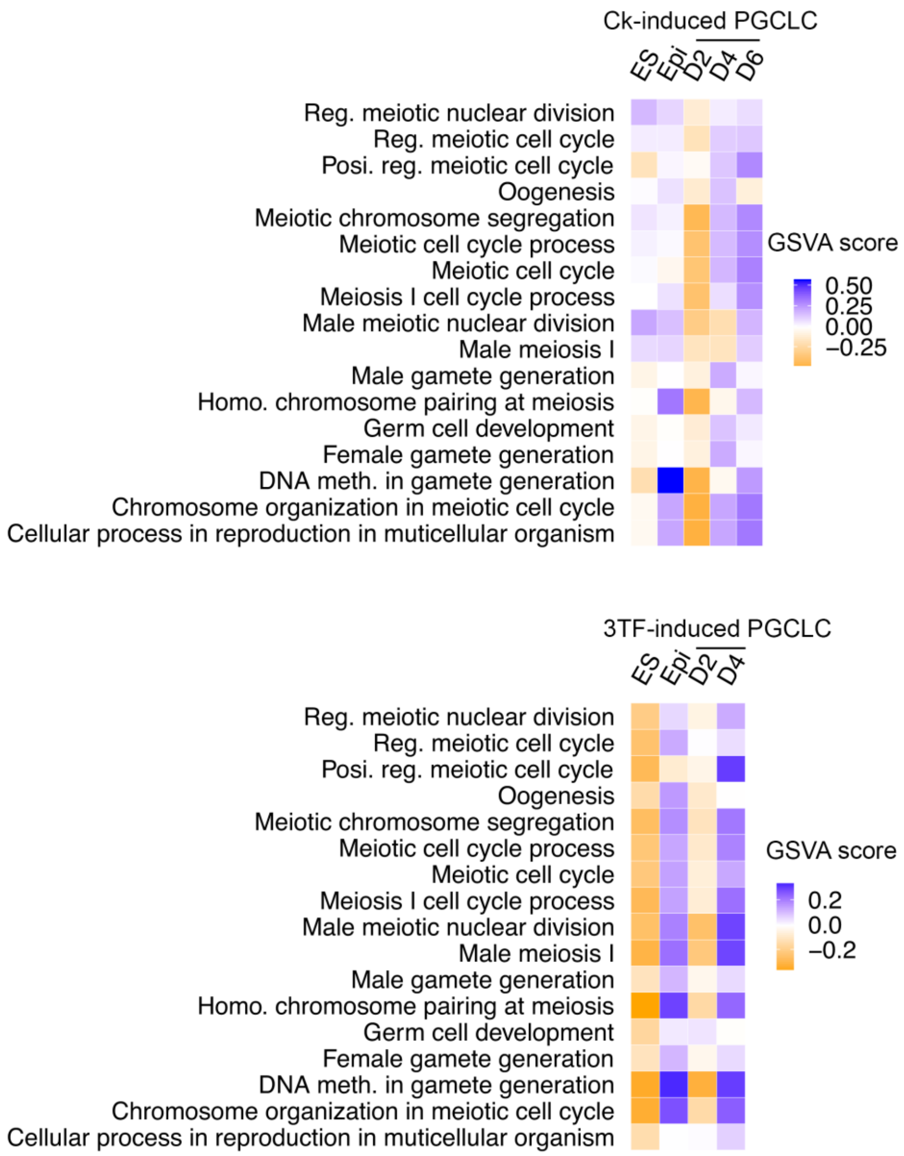

Appendix Figure S4. Heatmaps showing the average GSVA enrichment score of selected germ cell development pathways in Ck- and 3TF- induced PGCLCs.

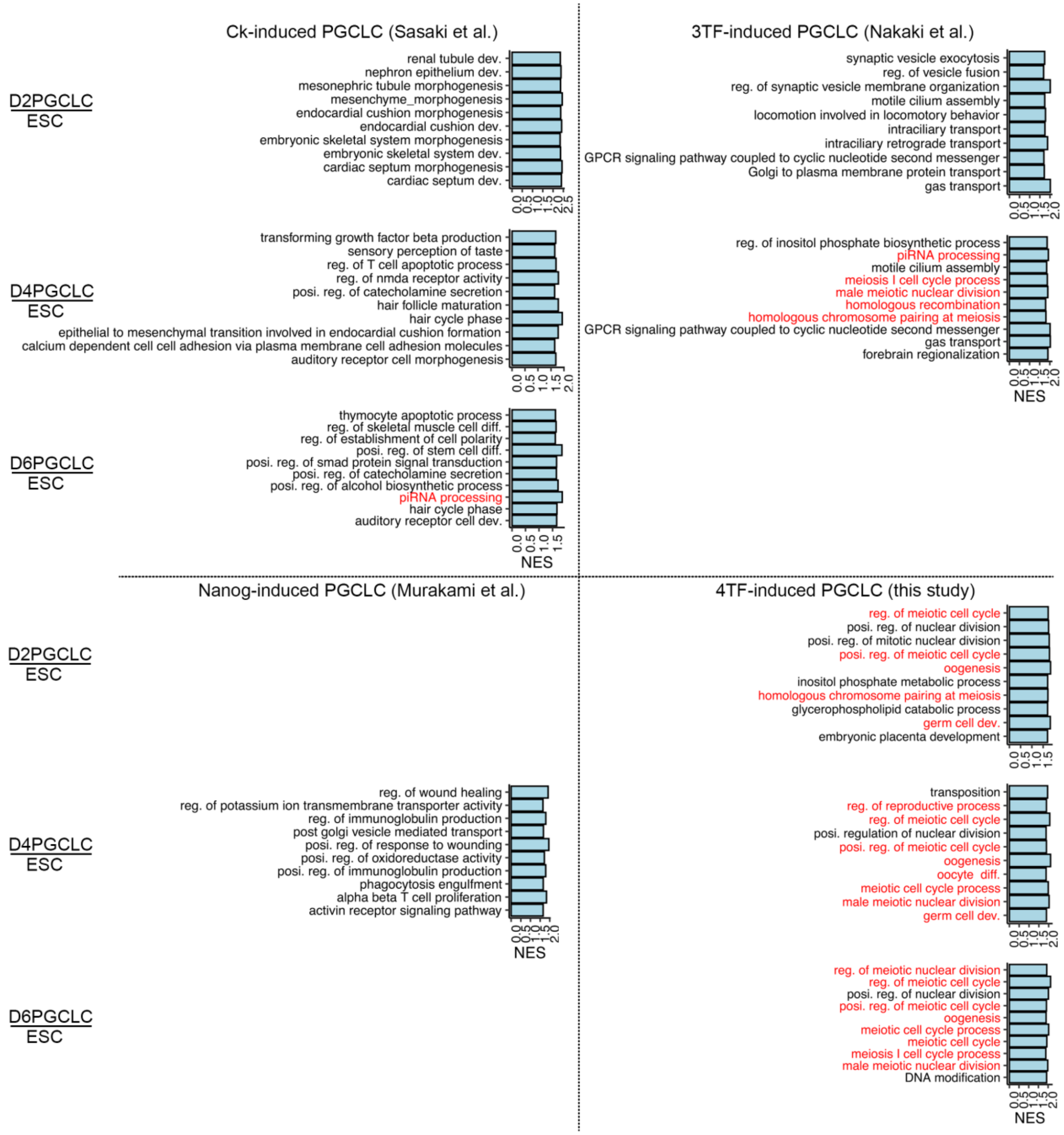

Appendix Figure S5. Top 10 enriched biological process GO terms in Ck-, 3TF-, Nanog- and 4TF-induced PGCLCs identified by GSEA. Terms of germ cell development (highlighted by red) was highly enriched in 4TF-induced PGCLCs. All the p-value of these GO terms were < 0.05. NES, normalized enrichment score.

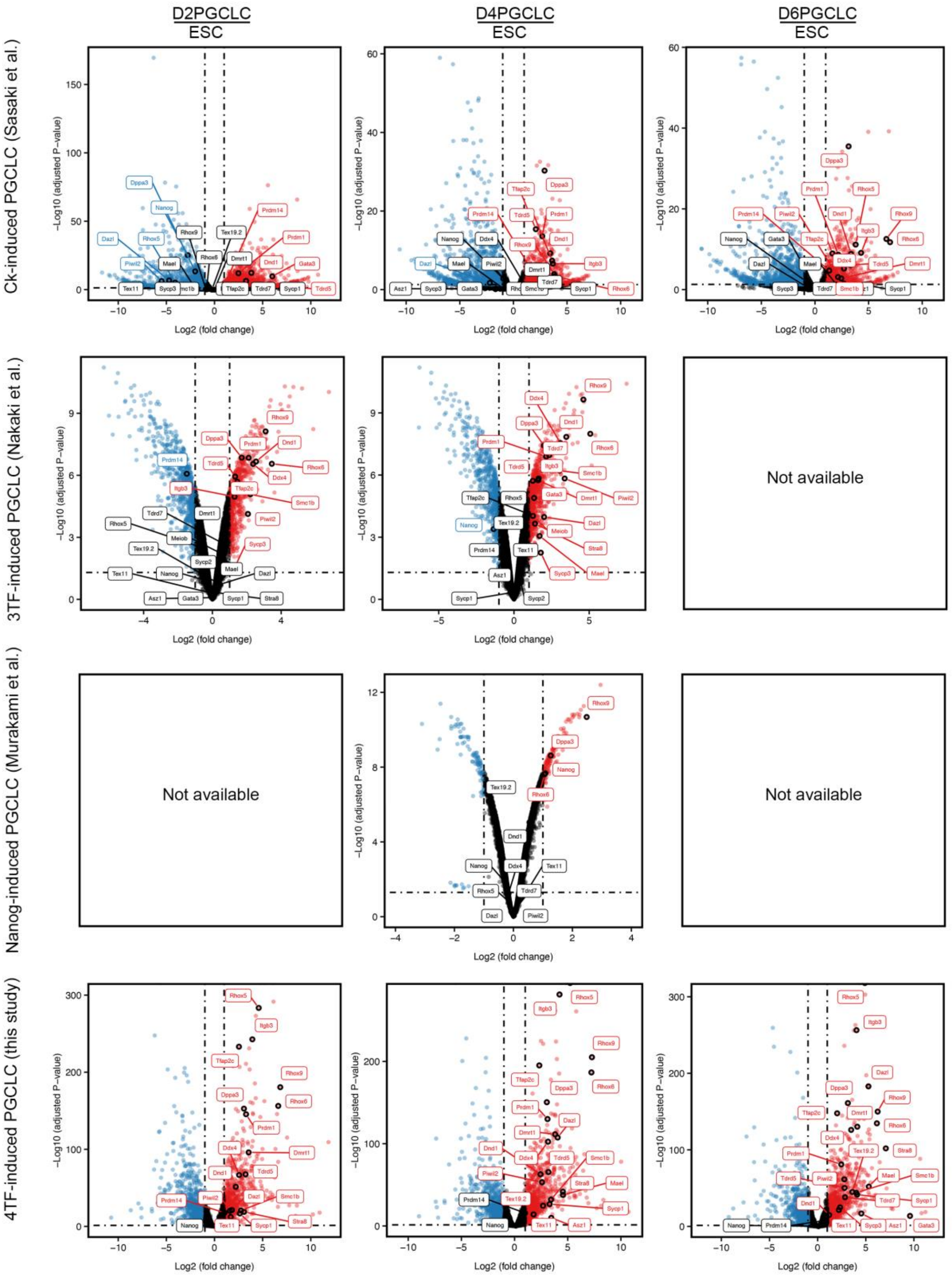

Appendix Figure S6. Volcano plot comparing transcriptomes of Ck-, 3TF-, Nanog- and 4TF-induced PGCLCs to ESCs. Dashed lines represent significance thresholds. Selected genes involved in germ cell development are highlighted.

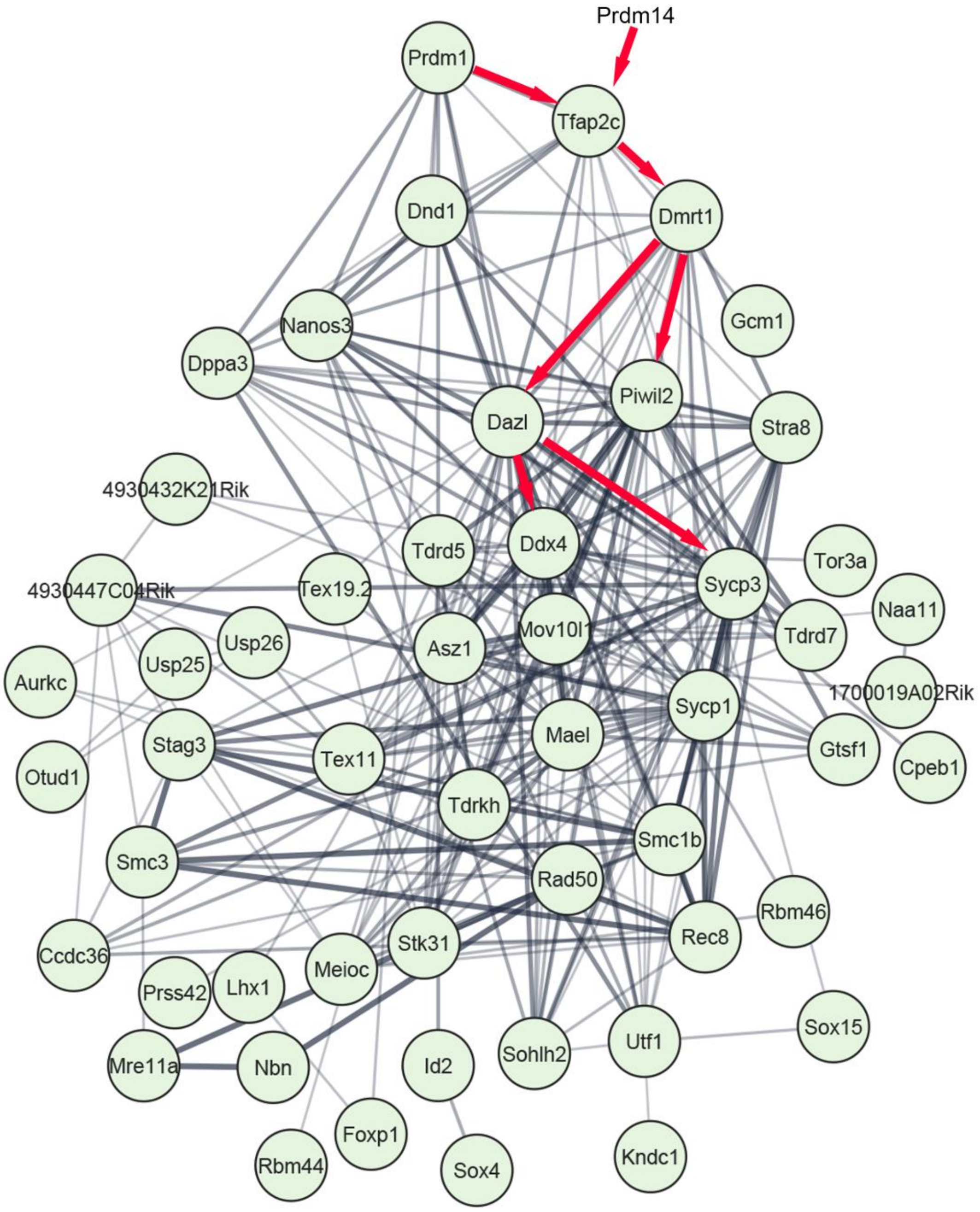

Appendix Figure S7. STRING analysis of upregulated DEGs in PGCLCs compared to ESCs. PRDM14 was omitted from the network construction because the log2(FC) of PGCLCs vs ESCs was <2. Red arrows indicate the transition from early to late PGCs.

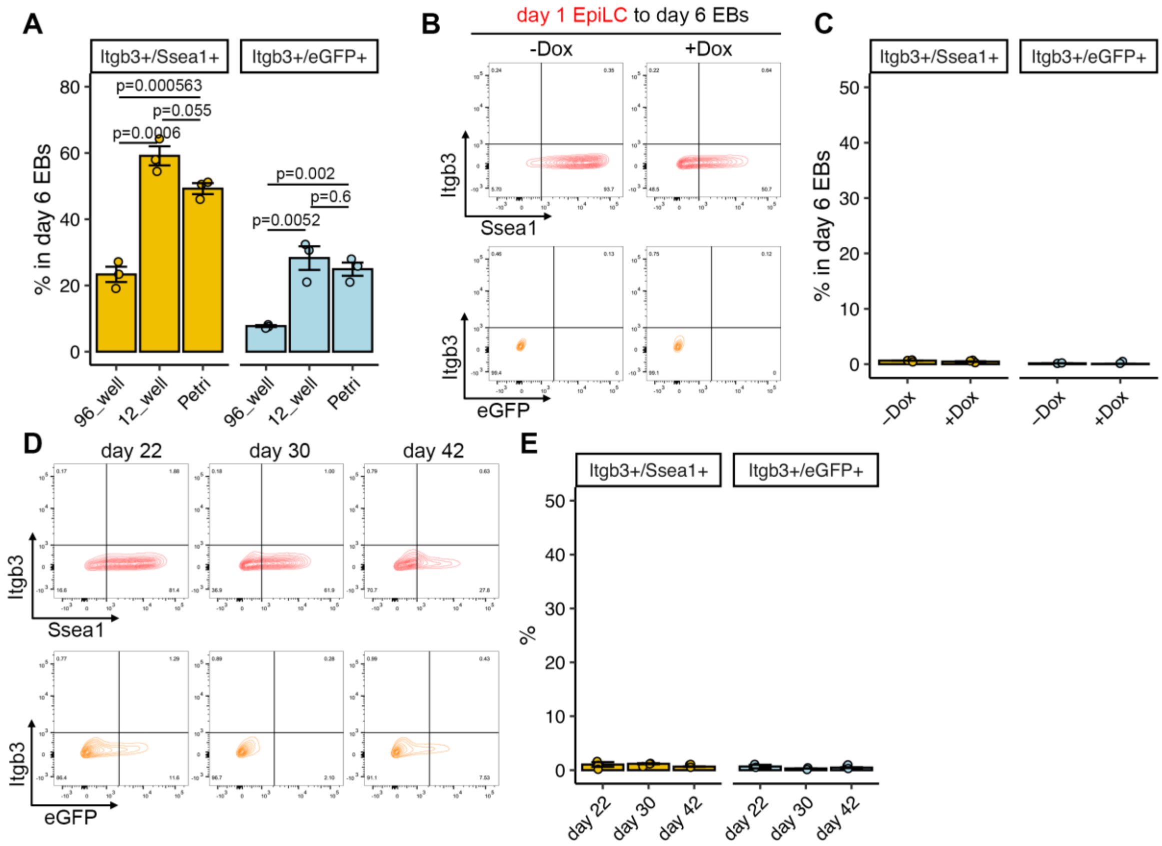

Appendix Figure S8. Differentiation of Nanog-induced PGCLCs. A. Quantification of PGCLC populations induced by Nanog in day 6 EBs at different plates/ dish. B. Representative FACS pattern of Itgb3^{+}/Ssea1^{+} and Itgb3^{+}/eGFP^{+} cells after inducing Nanog alone or not in day 1 EpiLCs for 6 days' suspension culture. C. Quantification of PGCLC populations in day 6 EBs with or without Dox treatment. D. Representative FACS pattern of Itgb3^{+}/Ssea1^{+} and Itgb3^{+}/eGFP^{+} cells after long-term culture in Nanog-inducible system. C. Quantification of PGCLC populations at different time points of long-term cultured EBs. Data in A, C and E are represented as the mean ± SEM. Data in A were analyzed using one-way ANOVA with Tukey's post hoc test.

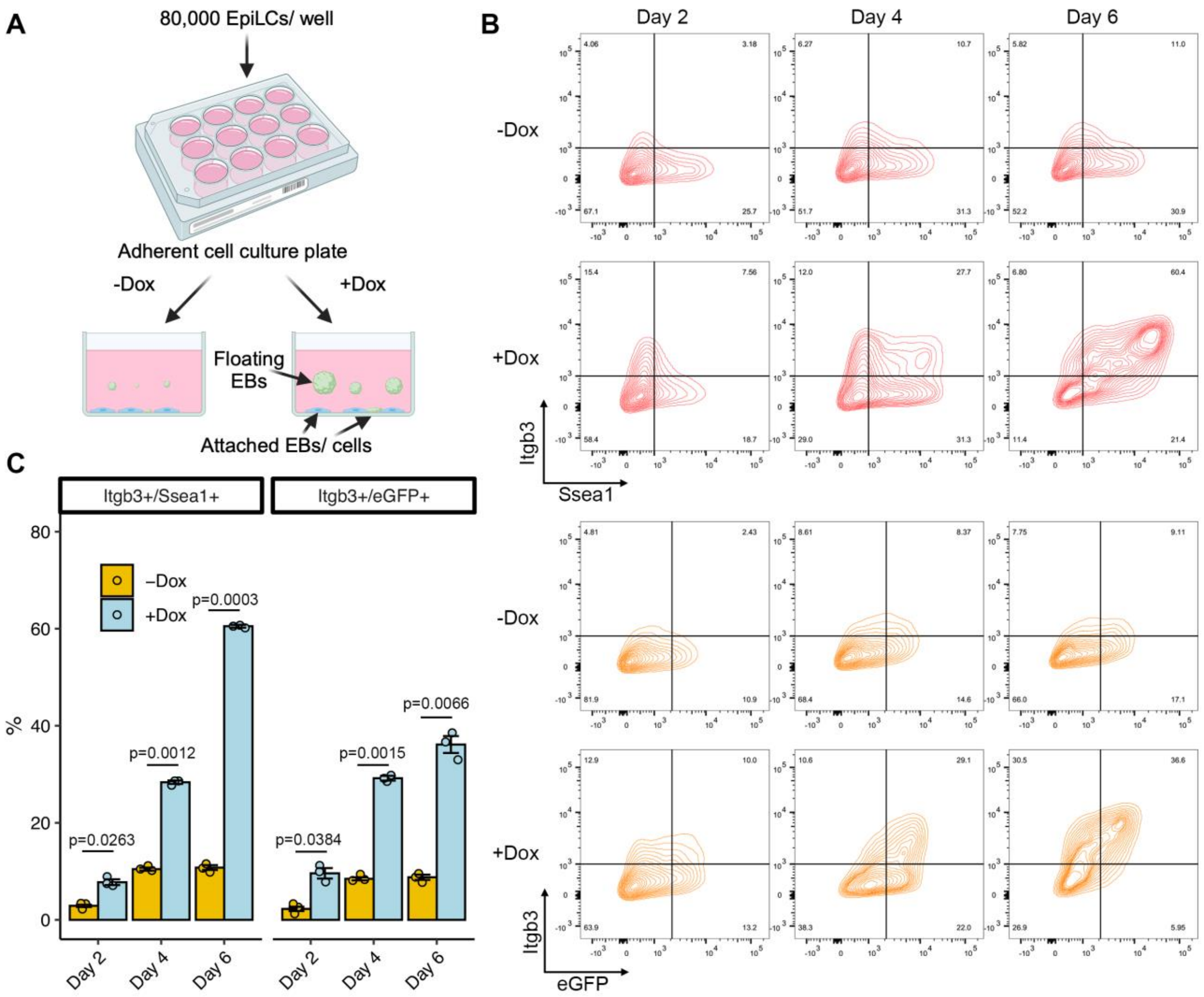

Appendix Figure S9. Differentiation of 4TF-induced PGCLCs in adherent cell culture plates. A. Schema of PGCLC differentiation; both floating EBs and attached cells were observed. B. Representative FACS pattern of Itgb3^{+}/Ssea1^{+} and Itgb3^{+}/eGFP^{+} cells after EpiLCs were treated by Dox or not during the 6-day period. The floating and attached cells were grouped for analysis. C. Quantification of PGCLC populations. Data in C are represented as the mean ± SEM and analyzed using a two-tailed paired t test.

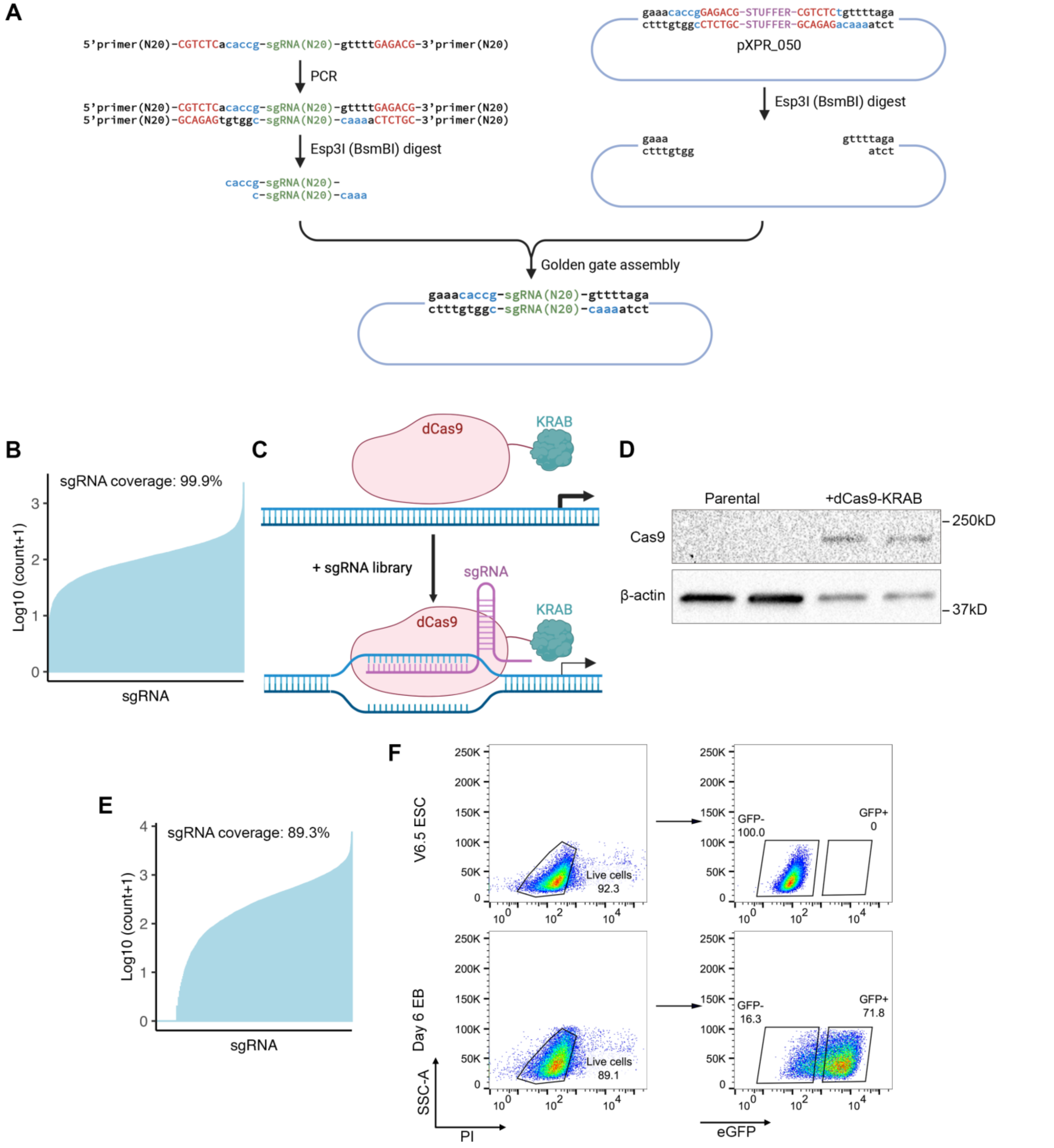

Appendix Figure S10. CRISPRi Library construction. A. Plasmid cloning of CRISPRi library. B. sgRNA coverage of the synthesized CRISPRi plasmid pool. C. Schematic illustration of CRISPRi using dCas9 and a KRAB repression domain programmed by sgRNA. D. dCas9 and beta actin protein levels of parental ESCs and ESCs stably transduced with dCas9-KRAB. E. Coverage of sgRNA vector integrations after viral infection into ESCs. F. FACS of Stella-eGFP^{+} and Stella-eGFP^{-} cells from day 6 dispersed EBs. PI, propidium iodide. V6.5 ESC, non-transgenic ESC line.

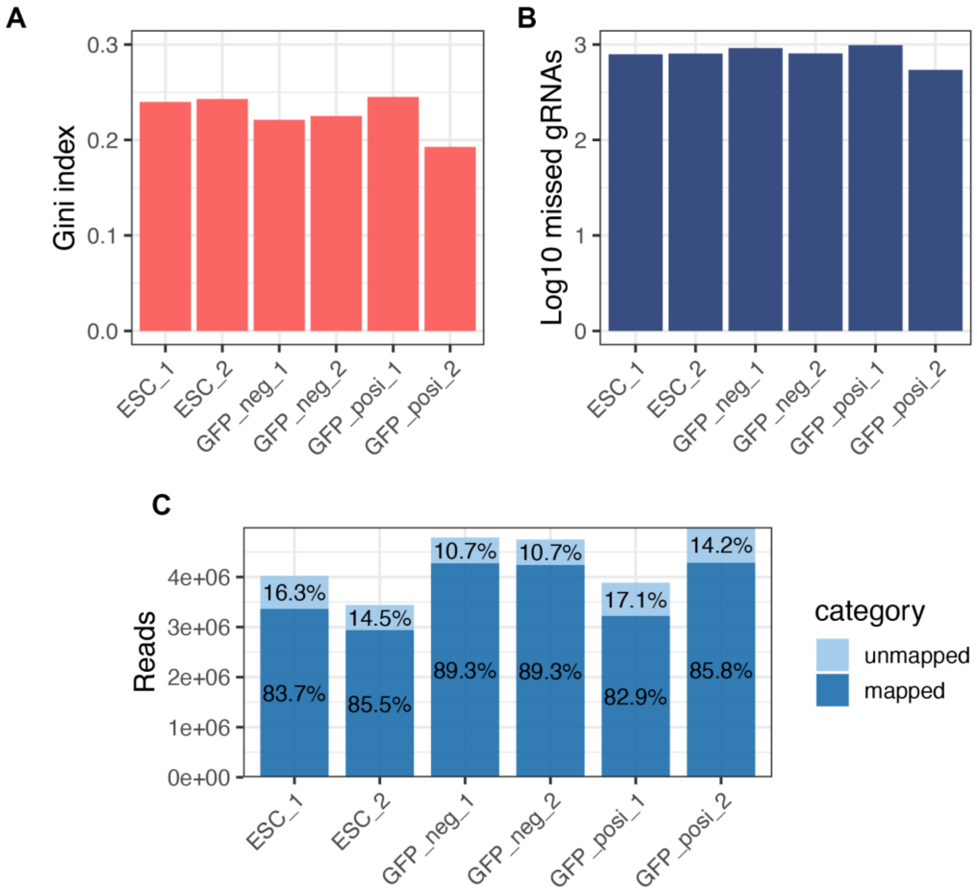

Appendix Figure S11. CRISPRi library characterization. (A) The Gini index measures the evenness of sgRNA read count. (B) Normalized number of missed sgRNA read count. (C) Number of reads and percentage of unmapped/mapped reads. All samples are from the CRISPRi screen dataset generated from ESCs, Stella-eGFP$^-$ (GFP_neg) and Stella-eGFP$^+$ (GFP_posi) cells. Data were generated using Screen Processing Tools pipeline.

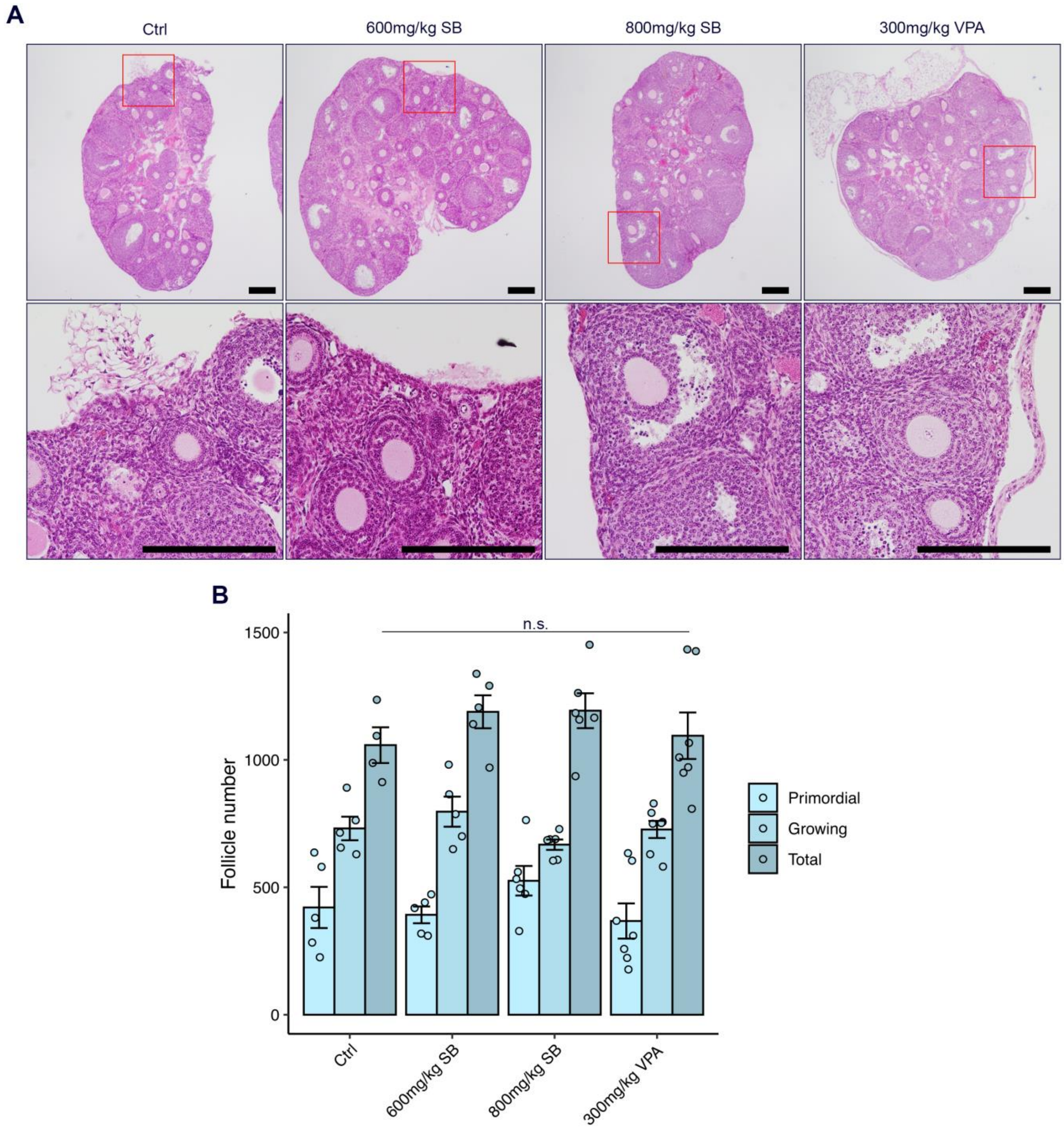

Appendix Figure S12. Phenotypic analysis of female pups. (A) Histological analyses of 1-month old ovaries. The boxed regions are magnifications of follicles showing in lower panel. Scale bars = 100 μm. (B) Follicle counts summed across every fifth serial section. Data are represented as the mean ± SEM and were analyzed using one-way ANOVA with Tukey's post hoc test. n.s. represents no significant difference.

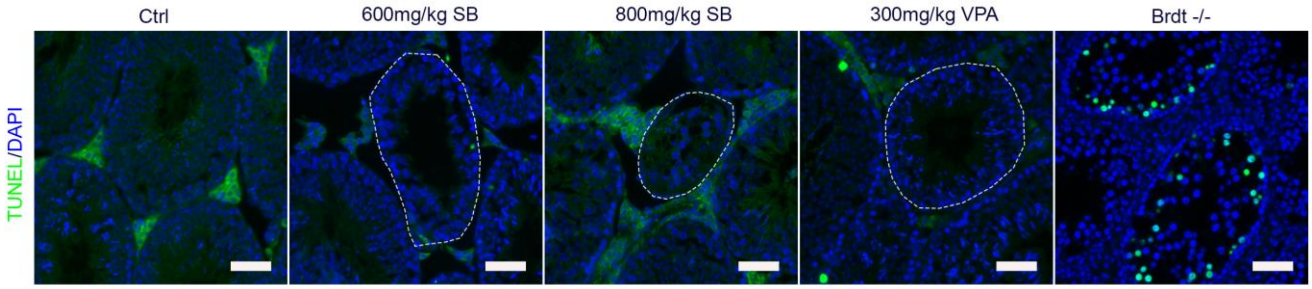

Appendix Figure S13. Absence of apoptotic cells in atrophic seminiferous tubules. Dashed lines denote regions with atrophic seminiferous tubules. Testicular section from $Brdt^{-/-}$ mouse model with a meiotic defect (Ding et al, 2023) was utilized as a positive control for TUNEL staining. Scale bar represents 50 μm.

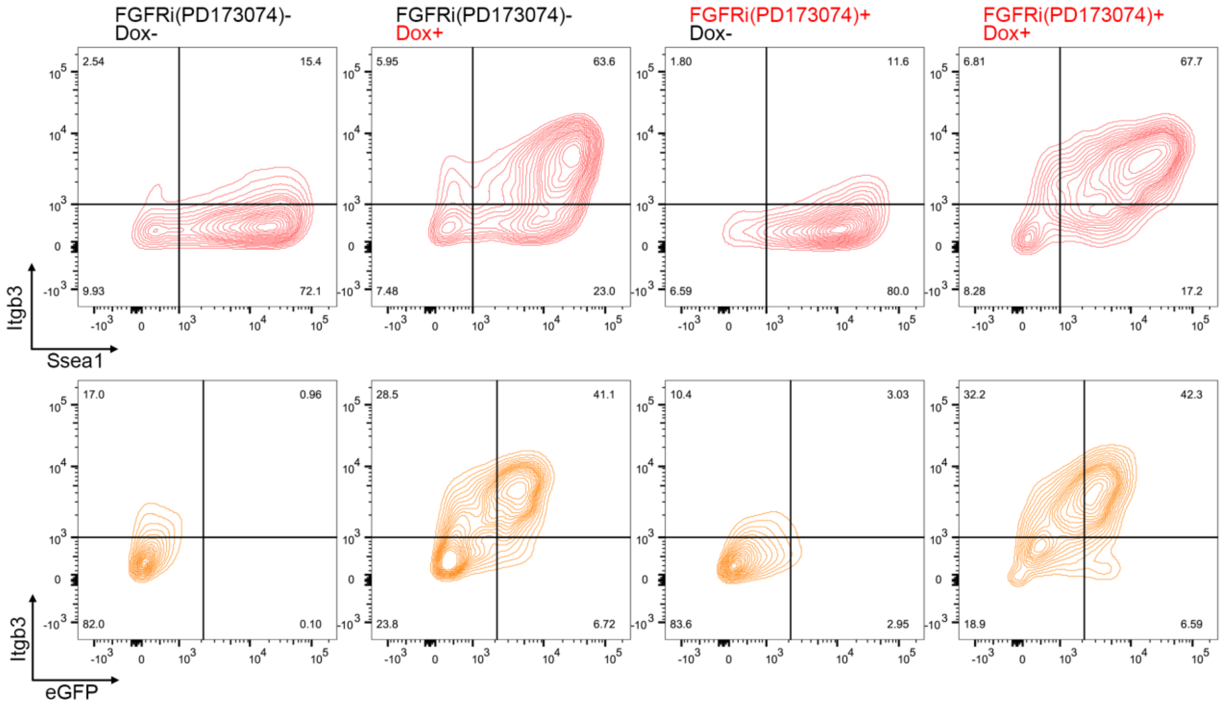

Appendix Figure S14. Impact of FGFRi on differentiation of 4TF-induced PGCLCs from formative ESCs. Day 6 cells from untreated 12-well plates were collected for FACS.

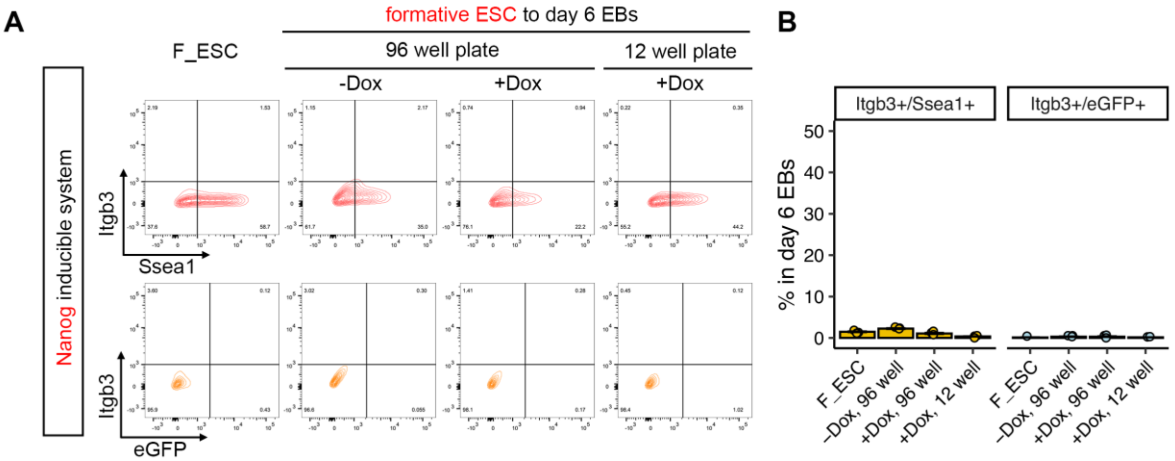

Appendix Figure S15. Overexpression of Nanog in suspended formative ESCs. A. Representative FACS pattern of Itgb3$^+$/Ssea1$^+$ and Itgb3$^+$/eGFP$^+$ cells in formative ESC and day 6 EBs from different plates and culture conditions. B. Quantification of PGCLC populations. Data in B are represented as the mean ± SEM.

	Ck	3TFs OE	Nanog OE	4TFs OE
High efficiency	No	No	No	Yes
Scalability	No	N.A.	Yes	Yes
Long-term PGCLC culture	N.A.	N.A.	No	Yes
Direct diff. from formative ESC	Yes	N.A.	No	Yes
Cost effectiveness	No	Yes	Yes	Yes

Appendix Figure S16. Comparation features of 4TF-inducible system with current available PGCLC differentiation systems. N.A., not available. The cost-effectiveness was determined based on the following analysis: preparing 10ml of PGCLC differentiation media using cytokines can cost up to $456 (based on small aliquots of BMP4). Considering the differentiation efficiency is far lower in cytokine-based protocols, generating an equivalent number of PGCLCs using cytokines would be over 500 times more expensive than using the 4TF system.
