# Supplementary material for: A primordial germ cell-like-cell platform enables CRISPRi screen for epigenetic fertility modifiers
Source: EMBO Rep. 2025 Nov 13;26(23):6044–78. doi: 10.1038/s44319-025-00633-z (PMC12678824; doi:10.1038/s44319-025-00633-z)
Supplement: Supplementary file 13 — Expanded View Figures [file 44319_2025_633_MOESM13_ESM.pdf]

Expanded View Figures

Figure EV1. Assessing the germline competence of Stella-eGFP ESCs and confirming NANOG's regulation of *Prdm1* and *Prdm14*.

(**A**) Chimeras generated by Stella-eGFP ESCs. (**B**) Representative of germline transmission of chimeras from Stella-eGFP ESCs. (**C**) Summary of chimeric mice generated by blastocyst microinjection. (**D**) Genotyping of pups to detect transgene originating from Stella-eGFP ESCs. (**E**) Scheme for CRISPR/Cas9-assisted transgene integration into the H11 "safe harbor" locus. (**F**) Overview of how ESCs were generated that contain NANOG-responsive reporters (*Prdm1* and *Prdm14* enhancers upstream of a *Shh* basal promoter) at the H11 locus. (**G**) PCR verification of clones harboring knock-in vectors. (**H**) Representative β-gal staining of EBs. (**I**) Schema illustrating key TFs acting during PGC development. Scale bar in (**H**) represents 50 µm. (**J**) Reverse transcription PCR of mRNAs in day 2 EBs.

© The Author(s)

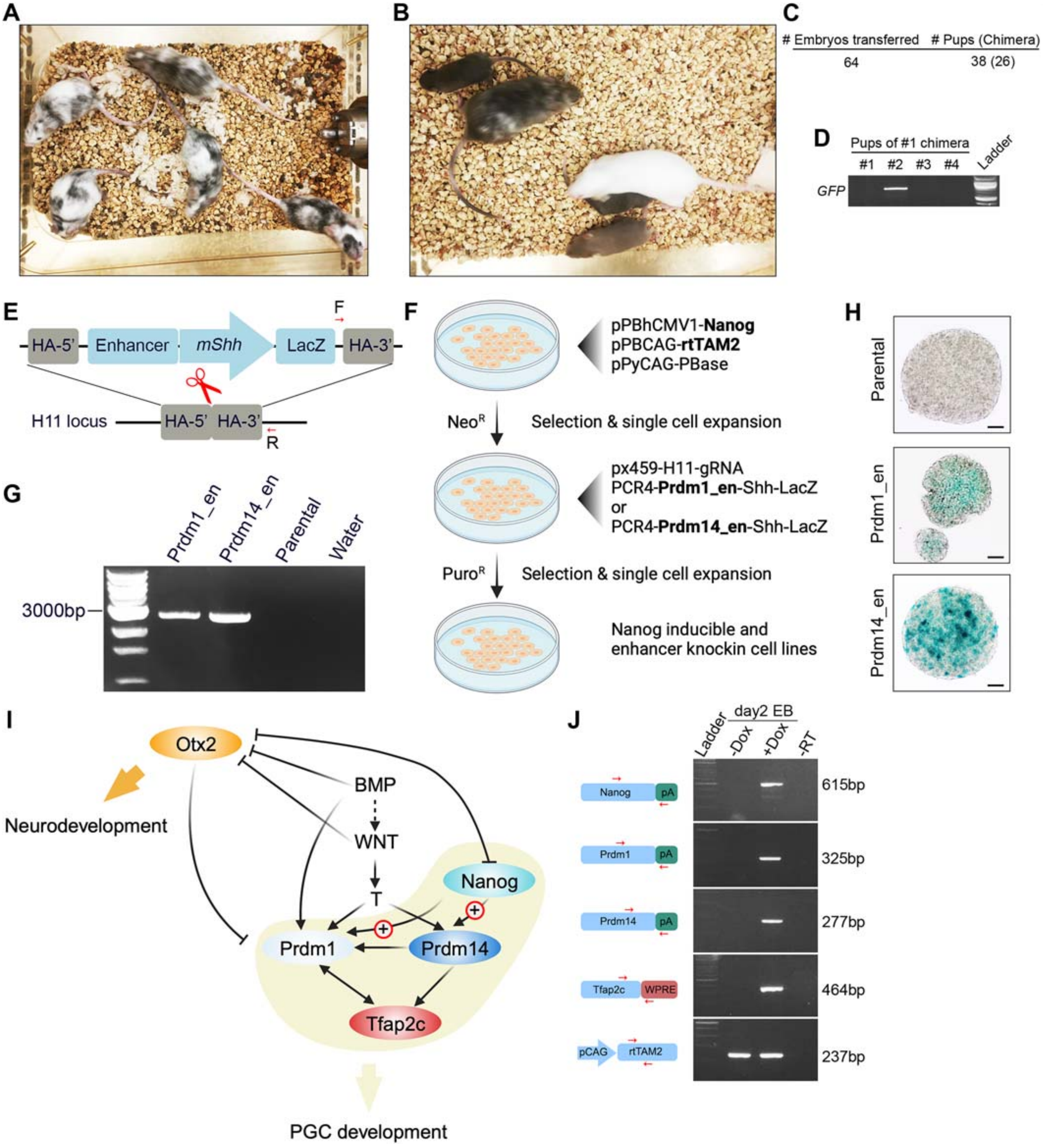
A
B
C
Embryos transferred
Pups (Chimera)
64
38 (26)
D
Pups of #1 chimera
#1 #2 #3 #4
Ladder
GFP
E
HA-5'
Enhancer
mShh
LacZ
HA-3'
F
R
H11 locus
HA-5' HA-3'
F
pPBhCMV1-Nanog
pPBCAG-rtTAM2
pPyCAG-PBase
NeoR
Selection & single cell expansion
px459-H11-gRNA
PCR4-Prdm1_en-Shh-LacZ
or
PCR4-Prdm14_en-Shh-LacZ
PuroR
Selection & single cell expansion
Nanog inducible and
enhancer knockin cell lines
G
Prdm1_en
Prdm14_en
Parental
Water
3000bp
H
Parental
Prdm1_en
Prdm14_en
I
Otx2
Neurodevelopment
BMP
WNT
T
Nanog
Prdm1
Prdm14
Tfap2c
PGC development
J
Ladder
day2 EB
-Dox
+Dox
-RT
Nanog
pA
615bp
Prdm1
pA
325bp
Prdm14
pA
277bp
Tfap2c
WPRE
464bp
pCAG
rtTAM2
237bp

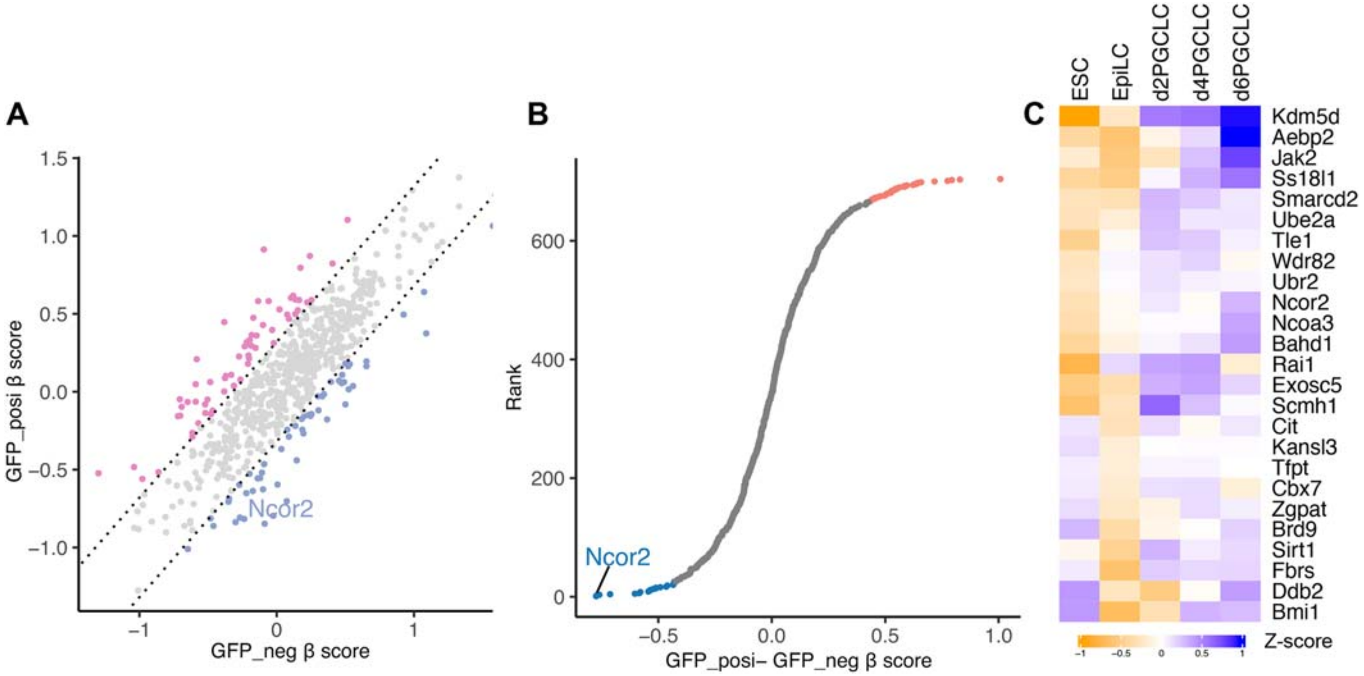

Figure EV2. CRISPRi screen epigenetic genes affecting PGCLC differentiation.

(**A**) Scatterplot of Stella-eGFP$^+$ and Stella-eGFP$^-$ β scores of each gene. The β scores were normalized using non-targeting sgRNA sequences. The two dashed lines indicates ± 1 S.D. of the differences between the Stella-eGFP$^+$ and Stella-eGFP$^-$ cells β scores. (**B**) Rank plot of the differential β scores, calculated by subtracting Stella-eGFP$^-$ β scores from Stella-eGFP$^+$ β scores. The color scheme of the dots is the same as in (**A**). (**C**) Gene expression heatmap of 25 candidate genes with β scores and exhibited increased expression during PGCLC differentiation compared to ESC and EpiLC stages.

 © The Author(s)

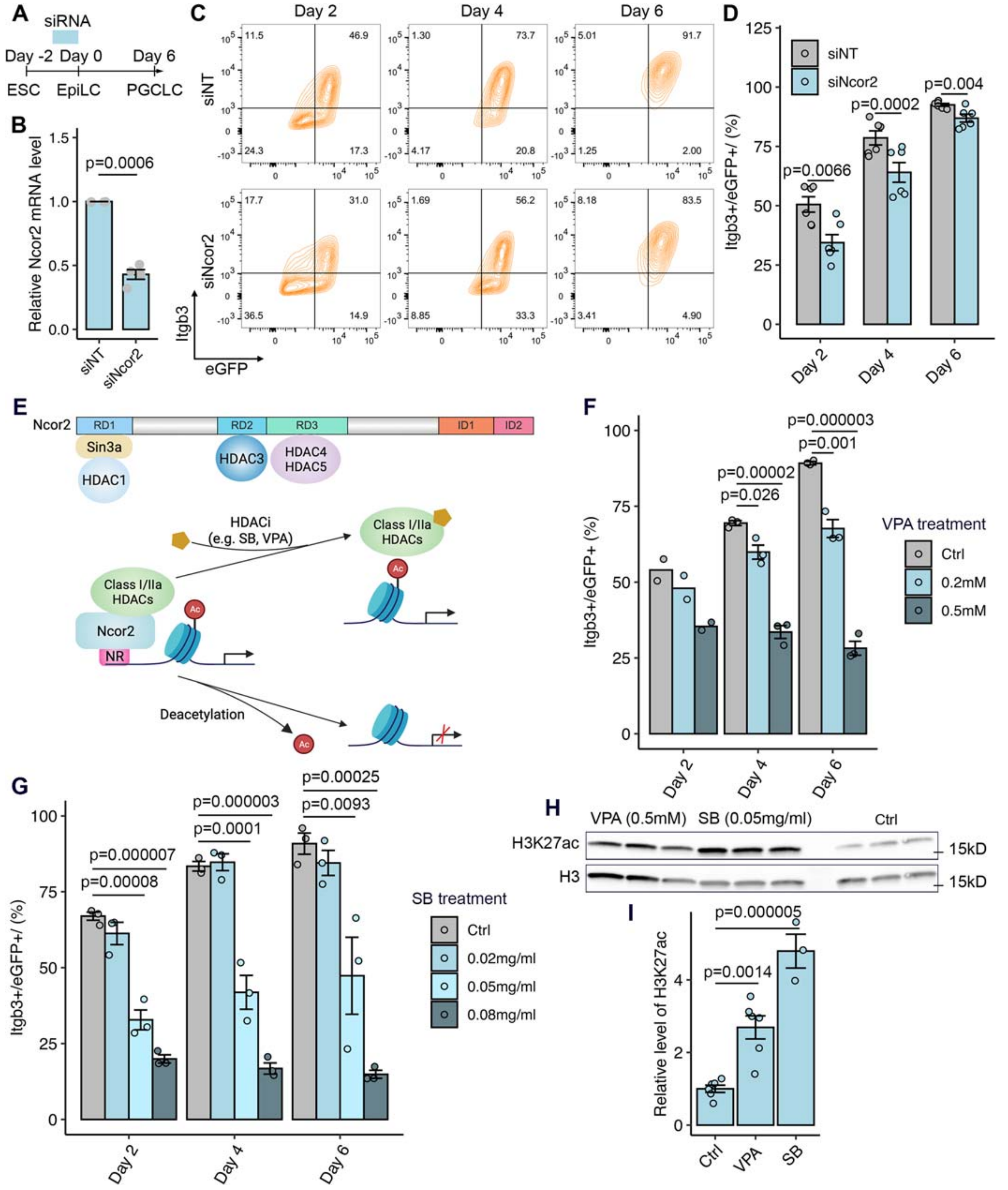
A
siRNA
Day -2 Day 0 Day 6
ESC EpiLC PGCLC
B
Relative Ncor2 mRNA level
p=0.0006
siNT
siNcor2
C
Day 2
Day 4
Day 6
siNT
siNcor2
Itgb3
eGFP
D
siNT
siNcor2
Itgb3+/eGFP+/ (%)
p=0.0066
p=0.0002
p=0.004
Day 2
Day 4
Day 6
E
Ncor2
RD1
RD2
RD3
ID1
ID2
Sin3a
HDAC1
HDAC3
HDAC4
HDAC5
HDACi
(e.g. SB, VPA)
Class I/IIa HDACs
Ncor2
NR
Ac
Deacetylation
F
Itgb3+/eGFP+ (%)
p=0.000003
p=0.001
p=0.00002
p=0.026
VPA treatment
Ctrl
0.2mM
0.5mM
G
Itgb3+/eGFP+/ (%)
p=0.000007
p=0.00008
p=0.000003
p=0.0001
p=0.00025
p=0.0093
SB treatment
Ctrl
0.02mg/ml
0.05mg/ml
0.08mg/ml
H
VPA (0.5mM) SB (0.05mg/ml) Ctrl
H3K27ac
H3
15kD
15kD
I
Relative level of H3K27ac
p=0.000005
p=0.0014
Ctrl
VPA
SB

Figure EV3. NCOR2 deficiency and HDAC inhibitors suppress PGCLC differentiation in vitro.

(**A**) Experimental schema showing siRNA treatment. (**B**) Effectiveness of *Ncor2* siRNA on day 2 after PGCLC induction. $n = 3$ biological replicates. mRNA levels quantified by qRT-PCR. siNT, non-targeting siRNA. (**C**) Relative population density in EBs treated with siNT or siNcor2. siNcor2, siRNA targeting *Ncor2*. (**D**) Percentages of Itgb3^{+}/eGFP^{+} cells in knockdown (siNcor2) vs control (siNT) groups at the indicated stages after transfection of small RNAs. $n = 6$ biological replicates. eGFP, Stella-eGFP. (**E**) Structure of NCOR2 and interaction with HDACs (upper panel). Impact of HDAC inhibitors on NCOR2-HDAC complexes (lower panel). (**F**) Percentage of Itgb3^{+}/eGFP^{+} populations within EBs treated with water (Ctrl) or VPA as assessed by flow cytometry. $n = 3$ biological replicates except there are 2 biological replicates for day 2. (**G**) Same as (**F**), except for SB treatment. $n = 3$ biological replicates. (**H**) Western blot analysis of H3K27ac and H3 protein levels in day 2 Stella-eGFP^{+} cells treated with HDACi. (**I**) Quantification of relative H3K27ac protein levels normalized to H3. $n \geq 3$ biological replicates. Data in (**B**, **D**, **F**, **G**, **I**) are represented as the mean ± SEM. Data in (**B**, **D**) were analyzed using a two-tailed paired *t* test, and data in (**F**, **G**, **I**) were analyzed using one-way ANOVA with Tukey's *post hoc* test.

© The Author(s)

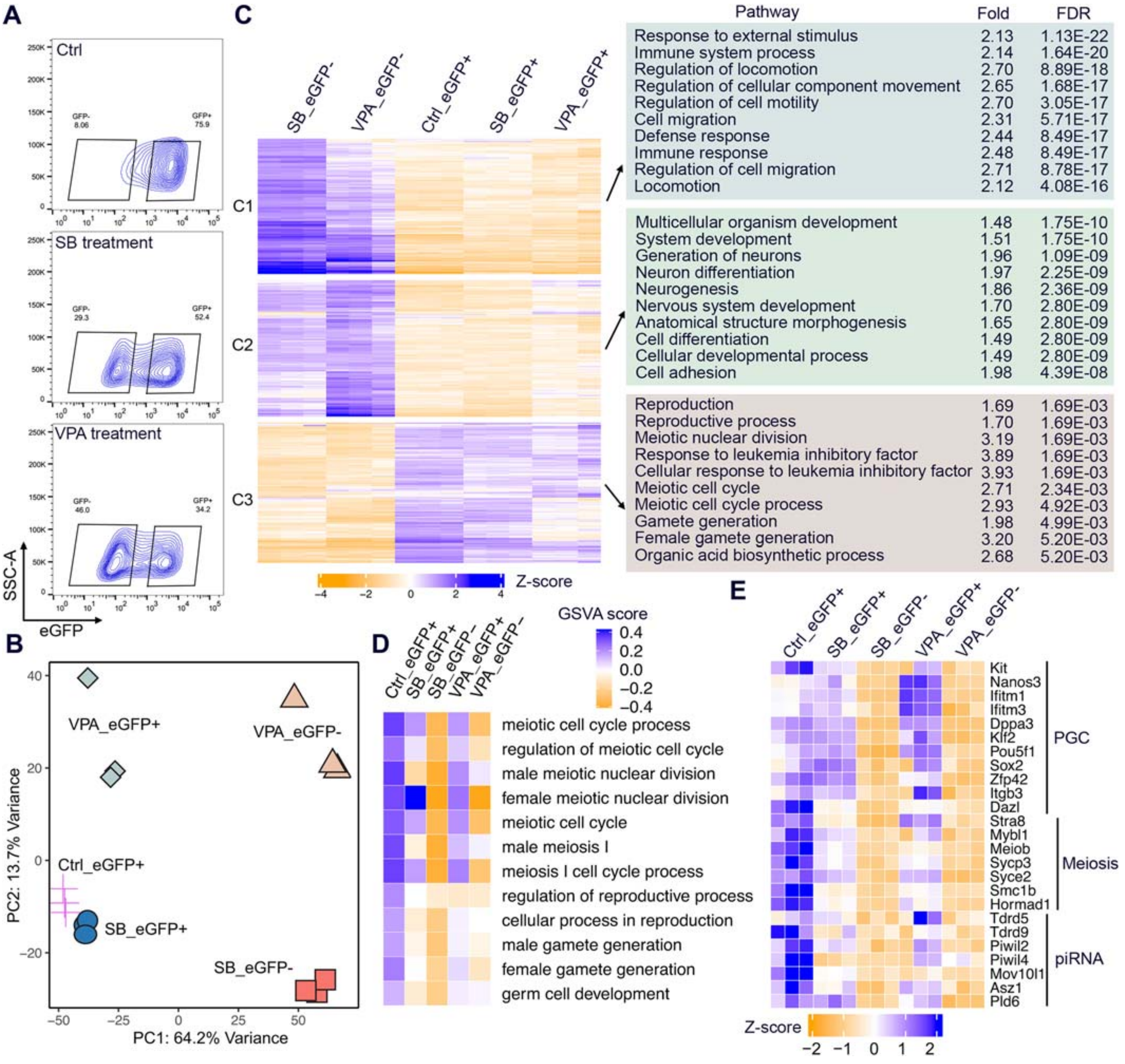

Figure EV4. Downregulation of germline networks by HDAC inhibitors.

(**A**) Flow sorting gates for Stella-eGFP+ and Stella-eGFP- cells from day 6 EBs used for RNA-seq. (**B**) PCA of transcriptomes of sorted cells of indicated groups. SB, sodium butyrate; VPA, valproic acid; Ctrl, control untreated. (**C**) Heatmap of k-Means clustering of variably expressed genes in SB, VPA and Ctrl cells ($n = 2{,}000$; $k = 3$). Genes were grouped into 3 clusters ("C1-3") based on expression similarity. Top enriched GO terms for the genes in each cluster shown with fold enrichment (Fold) and false discovery rate (FDR). (**D**) Heatmap showing the average GSVA enrichment score of selected germline development pathways. (**E**) Heatmaps of normalized RNA-seq reads for selected germline genes.

© The Author(s)

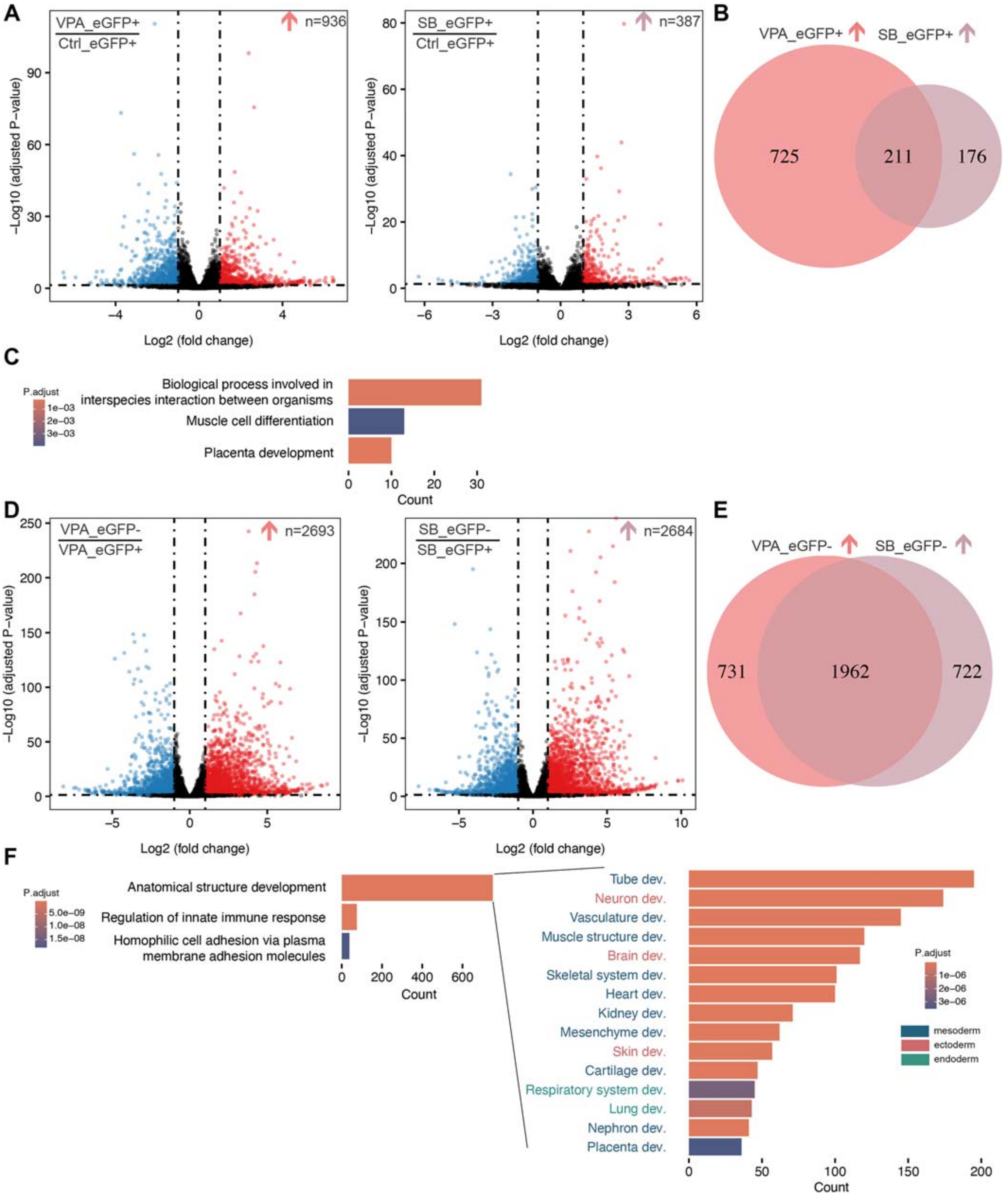
A
VPA_eGFP+
Ctrl_eGFP+
n=936
SB_eGFP+
Ctrl_eGFP+
n=387
−Log10 (adjusted P−value)
Log2 (fold change)
B
VPA_eGFP+
SB_eGFP+
725
211
176
C
P.adjust
1e−03
2e−03
3e−03
Biological process involved in interspecies interaction between organisms
Muscle cell differentiation
Placenta development
Count
D
VPA_eGFP-
VPA_eGFP+
n=2693
SB_eGFP-
SB_eGFP+
n=2684
E
VPA_eGFP-
SB_eGFP-
731
1962
722
F
P.adjust
5.0e−09
1.0e−08
1.5e−08
Anatomical structure development
Regulation of innate immune response
Homophilic cell adhesion via plasma membrane adhesion molecules
Count
Tube dev.
Neuron dev.
Vasculature dev.
Muscle structure dev.
Brain dev.
Skeletal system dev.
Heart dev.
Kidney dev.
Mesenchyme dev.
Skin dev.
Cartilage dev.
Respiratory system dev.
Lung dev.
Nephron dev.
Placenta dev.
P.adjust
1e−06
2e−06
3e−06
mesoderm
ectoderm
endoderm

 © The Author(s)

Figure EV5. Evidence of somatic lineage differentiation induced by HDACi treatment of differentiating PGCLC cultures.

(**A**) Volcano plots of log_2 (fold change) versus $-log_{10}$ (adjusted *P* value) for VPA_eGFP$^+$ (left) or SB_eGFP$^+$ (right) versus Ctrl_ eGFP$^+$ cells. (**B**) Venn diagrams of the upregulated DEGs from (**A**). (**C**) Top enriched GO terms for the commonly upregulated genes in eGFP$^+$ cells in HDACi treatment groups. (**D**) Volcano plots of Stella-eGFP$^-$ versus Stella-eGFP$^+$ cells treated with VPA (left) or SB (right). (**E**) Venn diagram of the commonly upregulated DEGs from (**D**). (**F**) Left: Top enriched GO terms for the HDACi upregulated genes from (**E**). Right: Count distribution of the "child" GO terms under "Anatomical structure development".

© The Author(s)
